# Autonomous Exploration and Map Construction of a Mobile Robot Based on the TGHM Algorithm

**DOI:** 10.3390/s20020490

**Published:** 2020-01-15

**Authors:** Shuang Liu, Shenghao Li, Luchao Pang, Jiahao Hu, Haoyao Chen, Xiancheng Zhang

**Affiliations:** 1 School of Mechanical and Power Engineering, East China University of Science and Technology, Shanghai 200030, China; shuangliu@ecust.edu.cn (S.L.); y30170354@mail.ecust.edu.cn (S.L.); y30170357@mail.ecust.edu.cn (L.P.); y45170022@mail.ecust.edu.cn (J.H.); xczhang@ecust.edu.cn (X.Z.); 2 Harbin Institute of Technology Shenzhen, School of Mechanical Engineering and Automation, Shenzhen 518055, China

**Keywords:** LIDAR detection, space exploration, topology, simultaneous localization and mapping

## Abstract

An a priori map is often unavailable for a mobile robot in a new environment. In a large-scale environment, relying on manual guidance to construct an environment map will result in a huge workload. Hence, an autonomous exploration algorithm is necessary for the mobile robot to complete the exploration actively. This study proposes an autonomous exploration and mapping method based on an incremental caching topology–grid hybrid map (TGHM). Such an algorithm can accomplish the exploration task with high efficiency and high coverage of the established map. The TGHM is a fusion of a topology map, containing the information gain and motion cost for exploration, and a grid map, representing the established map for navigation and localization. At the beginning of one exploration round, the method of candidate target point generation based on geometry rules are applied to extract the candidates quickly. Then, a TGHM is established, and the information gain is evaluated for each candidate topology node on it. Finally, the node with the best evaluation value is selected as the next target point and the topology map is updated after each motion towards it as the end of this round. Simulations and experiments were performed to benchmark the proposed algorithm in robot autonomous exploration and map construction.

## 1. Introduction

The studies of robotics cover a wide range of research directions. The applications of robot localization, navigation, and path planning are founded on a well-established map of the operating environment. In a large-scale unknown environment, exploration and map construction based on human guidance is cumbersome. Therefore, a key issue in robot research is the study of how robots can efficiently and reliably explore an unknown environment and construct the environment map. Equipped with range sensors or visual sensors, a robot can carry out a systematic exploration without knowledge of the layouts of its surroundings or the arrangement of the obstacles.

The state-of-the-art exploration methodologies aim to find solutions for one single robot or a group of robots. While ensuring the integrity of the environment map, the efficiency of the strategy is most considered in exploration tasks. Under the premise of applying the same path planning method, proper strategies can avoid redundant movements carried out by unnecessarily generated target points and thus dramatically reduce the traversable cost.

In order to achieve an efficient strategy for one single robot, this study proposes a robot automatic exploration algorithm based on an incremental caching topology–grid hybrid map (TGHM). Such an algorithm is capable of establishing an unknown environment map with fewer target points and lower time consumption. Geometry rules are used to generate candidate target points in the active area [1]. Then, each candidate target point is evaluated on the basis of the surrounding unknown area. Each candidate target point that meets the evaluation criteria will be included in the topology map as a candidate topology node. The evaluation criteria consider information gain and motion cost. The node with the best evaluation value will be selected as the next target point. The topology map is updated along with each action instantly. By using the topology–grid hybrid map, the exploration strategy is well-organized and automatically fits the area under exploration. 

The contributions of this study fall into the following perspectives:

1. A geometry rule-based method for selecting candidate target points in an active area is proposed. The geometry rule is based on sensor information. This method can generate more candidate target points than the forward simulation (FS)-based method and only considers the frontiers in the active area for increasing computation efficiency.
2. The algorithm adopts TGHM to provide global planning for exploration strategies. The topology map records all past target points and candidate target points that have not been visited. When the mobile robot achieves the target position and starts the next exploration round, the criteria value of each candidate target point is evaluated on the basis of the motion cost provided by the topology map. Moreover, the information gain around each candidate target point provided by the grid map is used to evaluate the value of each point. Finally, the candidate target point with the best value will be selected as the next target point.
3. Simulations and experiments were performed to demonstrate the proposed method for autonomous exploration. The results proved that such a method can effectively optimize exploration efficiency. Moreover, a topology map can help prevent the mobile robot from repeatedly exploring the previously explored areas, thereby largely reducing the exploration cost. With the help of a grid map, navigation is executed on the optimal path.

This paper is organized as follows: Section 2 reviews the related work and previous studies of robot exploration. Section 3 describes the framework of the proposed algorithm. Section 4 introduces the method of selecting the candidate target by scanning the frontier. Section 5 presents the method of evaluating candidate points along with the construction and update of the topology–grid hybrid map. Section 6 provides detailed description and examples for the proposed exploration algorithm. Section 7 discusses the experiments conducted in both simulations and real-world environments to benchmark the efficiency of the TGHM against state-of-the-art methods in robotics automatic exploration. Section 8 provides a conclusion and discusses future works.

## 2. Related Work

This section presents the related technology necessary for executing robot exploration and reviews the previous works.

### 2.1. Related Technology

Simultaneous localization and mapping (SLAM) [2,3] is a common technology for exploring an unknown environment. According to the sensors applied for sensing the surrounding environment and the dimensions of the observation information, SLAM can be divided into 2D or 3D systems. The 2D-SLAM systems are equipped with range sensors like LIDARs or SONARs. Along with the odometer data uploaded by the mobile platform, the systems are capable of estimating the robot position and establishing 2D maps representing the structure and obstacle arrangement with probabilistic algorithms.

Map quality has a great influence on goal pose selection and path planning. Thus, an appropriate algorithm must be selected to construct a map. A popular method is EKF SLAM, which applies the extended Kalman filter to solve the nonlinear problems. However, the Gaussian noise assumption limited its performance in dealing with uncertainty [4]. KartoSLAM [5], based on graph optimization has advantages in a small environment. Stachniss et al. [6] studied myopic exploration in RBPF SLAM [7] by discretizing the action space to a set of possible waypoints and evaluating the approximate expected information gain when traveling to the waypoints by sampling. This work was later expanded by considering the alternative measures of uncertainty of SLAM solution [8,9]. These approaches have considered the exploration of new areas and the maintenance of the consistency of particle filter [10] approximation. 

In order to navigate a robot with less calculation load, a 2D grid map, or an occupancy grid map, is often generated according to the established environment map for robot localization and navigation purposes [11]. As a metric representation, it is a simplified but comprehensive description of the environment in a defined coordinate [12]. In exploration tasks, the grid map refines the drivable area for the path planning algorithms to generate an optimal path. It consists of three types of basic cells:

1. Occupied cells mark the area that is unreachable for the existence of obstacles or structural barriers.
2. Free cells mark the area that is explored and free for the robot to move.
3. Unknown cells mark the area that is unknown to the robot and contains information gain for exploration.

The costmap [13], a form of occupancy grid, is such a configurable structure that provides the robot information essential for navigation. Along with the robot data including size, footprint, safe distance, orientation, and so on, the costmap can provide the robot with a simplified but effective map to plan the optimal path. 

Path planning can be applied by Dijkstra’s [14], A* [15], and D* algorithms [16]. Dijkstra’s algorithm can derive the optimal solution of the shortest path, but it is inefficient because it traverses excessive nodes [17]. A* algorithm is based on a depth-first search, and its calculation cost is considerably lower than that of Dijkstra’s algorithm. D* algorithm is more suitable for dynamic networks than the other two algorithms. In this paper, A* is selected as the local path planning algorithm while the proposed TGHM algorithm is responsible for the global target point selection in exploration.

### 2.2. Previous Work on Exploration

The equipment required for accomplishing a robot exploration task is listed as the following [18]:

1. A mobile robot: It provides the odometer data and robotics characteristics necessary for establishing the motion model.
2. A computing unit: It provides resources for the algorithm to run. CPU, RAM, clock frequency, and other units should be considered to match the calculation requirement of the algorithm to achieve better performance.
3. Sensors: They provide sensing data from the environment. LIDAT, SONAR, RGB-D, and STEREO are widely applied in robotics research. The sensor precision, frequency, range, and other characteristics can impact the exploration and mapping quality.

To explore an unknown environment, the robot must possess the ability to decide where to navigate according to a specified strategy. A common exploration strategy is to obtain candidate goal points at the frontier [19] and then evaluate these candidate goal points on the basis of the utility function to select the optimal target point [20]. The reasonability and robustness of frontier detection have a great influence on this strategy. The frontier region on the grid map is often recognized using digital picture processing techniques [21,22]. However, the calculation of frontier detection and path planning on the grid map will rapidly increase as the extension of the exploration area. Therefore, the efficiency of mobile robot exploration will be significantly decreased. In addition, the frontier-based method always selects the nearest candidate target point, which limits the performance of the target point selection to a constrained area.

Freda [23] proposed a hybrid algorithm under the premise of frontier theory. This algorithm randomly generates observation poses and directs sense poses toward the unexplored environment. Oriolo [24] presented a search strategy, namely, a sensor-based random tree. In this algorithm, the next exploration node is a randomly selected boundary around the current node. Tae-Bum Kwon and Jae-Bok Song introduced a thing-based topological exploration strategy [25] based on the real-time construction of topological map nodes in the map. The robot determines whether or not to explore a node by analyzing the sensor data.

Recently, a forward simulation (FS)-based autonomous exploration algorithm has been proposed [26]. This method initially applies sequential Monte Carlo planning in order to generate random potential paths and then computes their reward values. After several iterations, the one with the highest reward value is chosen as the output. Several points through the path can be selected as the local target for the mobile robot. The FS algorithm managed to achieve higher accuracy and better stability than the utility function. However, this algorithm is time-consuming, especially for iterative calculations, and the length of the simulated paths is often limited. These drawbacks lead to low efficiency, especially in a large environment. In addition, the FS algorithm lacks a global strategy, which causes the mobile robot to access the same area at various times repeatedly.

The abovementioned methods can realize the traversal of an unknown environment. However, motion cost and path optimization are ignored when the next exploration point is determined. Thus, exploration is not optimal or efficient. Ge et al. [27,28,29,30] used a hierarchical topology map to represent the global environment information for improving the exploration efficiency of the robot. Moreover, the environment exploration algorithm of simultaneous path planning and topology mapping is realized. These methods avoid the frontier detection problem based on the grid map, but they generate unnecessary calculations with target points in non-frontier regions. In addition, the navigation of the mobile robot is based on the topology map, such that the path planning of the mobile robot is not the optimal path, which increases exploration cost. 

Aiming to increase the efficiency of the robot exploration, the proposed TGHM algorithm applies a geometry-rules based method to avoid the computationally expensive potential paths selection in the first place. Then the global exploration strategy is well organized by an incrementally updated topology map to generate the optimal target point for each exploration stage. The information gain and motion cost are taken as the criteria for target point selection, ensuring the balance between coverage and efficiency of the exploration.

To evaluate a map established by the exploration algorithm, the most common way is to calculate the difference between the output map and the ground truth map [31]. Thus, the completeness, or coverage rate, of the built map is a major metric for exploration [18]. For efficiency, the time consumption to complete an exploration task is regarded as another metric [32]. In addition, the number of target points in exploration and the traveled path length of the robot are also considered as metrics in this paper.

## 3. Algorithm Framework

**Figure 1:**
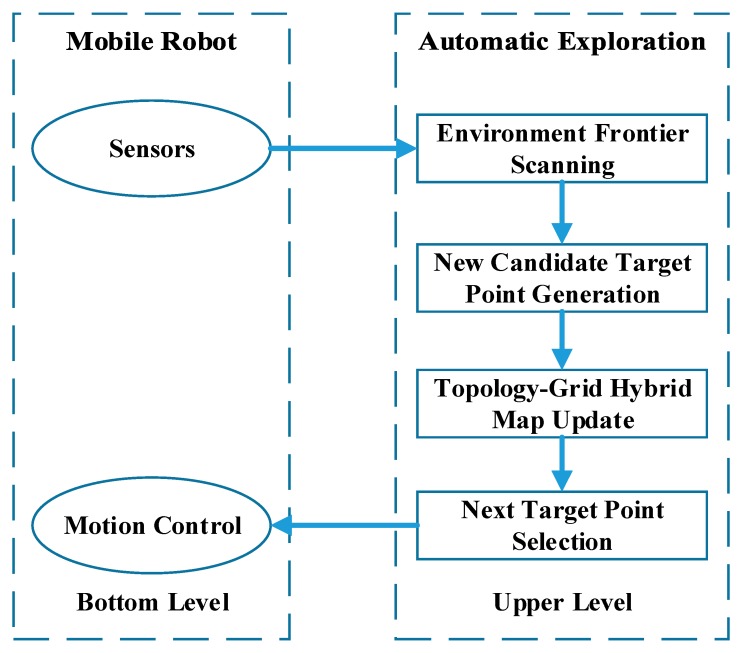
Topology–grid hybrid map (TGHM) algorithm framework.

As shown in Figure 1, the framework of the proposed TGHM algorithm consists of two levels:

1. The bottom level is where the robot collects sensor data, including the LIDAR scan data and robot odometer information for grid map establishment and information gain calculation. Once the bottom level achieves the outputted target point location from the upper level, it executes the motion to reach the target point generated by the exploration algorithm.
2. The upper level contains four processes to carry out the automatic exploration, including environment frontier scanning, new candidate target points generation, topology–grid hybrid map update, and next target point selection.

In more detail, the button level applies the GMapping algorithm to perform SLAM [33]. This layer computes the pose of the robot and creates the occupancy grid map on the basis of the rangefinder and odometer data. For each round of automatic exploration, the upper level executes five specific steps to decide the next target point:

1. The environment frontiers are detected in the active exploration area, and candidate target points are generated on the basis of environment geometry rules.
2. These newly generated candidate target points are added to the topology map as candidate topology nodes.
3. The candidate topology node that does not meet the requirements will be removed.
4. Each candidate topology node is evaluated and sorted on the basis of information gain and motion cost.
5. The candidate topology node with the highest value will be chosen as the next target point, and the node will turn into a topology node.

Once the next target point is determined, the motion control module is responsible for planning the path and move the robot towards it.

Below are the corresponding definitions of the terms used in this study.

- Candidate target point: A point generated on the basis of environment geometry rules.
- Candidate topology node: A node transformed from the qualified candidate target point.
- Topology node: A node transformed from the visited candidate topology node.
- Next target point: The target point for exploration in the next robot action.

## 4. Geometry Rules for Candidate Target Point Generation

**Figure 2:**
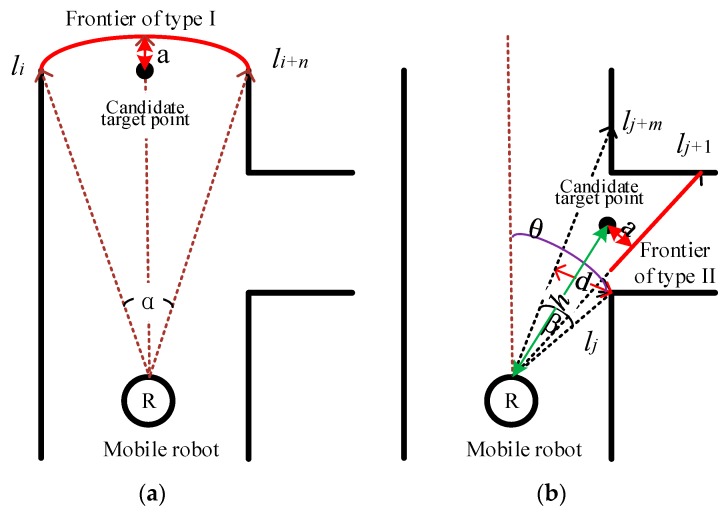
Two types of frontiers and the generation of candidate target points: (**a**) Frontier of type I; (**b**) Frontier of type II.

The candidate target point is generated on the basis of the environment frontier. The area within the sensor’s scan range can be regarded as the active exploration area. The environment frontiers of the active area consist of two types in this work as shown in Figure 2. *R* indicates the location of the robot and sensor. The ray of the sensor is represented as the dotted arrow. The environment frontier recognized on the basis of sensor data is marked as the solid line.

Frontier type I is at the top range of the sensor. This recognition rule of frontier type I is as follows:

(1) $$l_{i,i+1,\ldots ,i+n}=\;l_{max},$$

where $l_{i,i+1,\ldots ,i+n}$ represents a continuous measurement signal from $l_{i}$ to $l_{i+n}$ and $l_{max}$ is the measurement limit. Equation (1) shows that frontier type I is generated at the limit of the sensor’s measurement. *n* must be sufficiently large to ensure that the mobile robot could pass the frontier safely. Thus, *n* can be calculated from

(2) $$\sqrt{l_{i}^{2}+l_{i+n}^{2}-2l_{i}^{\;}l_{i+n}^{\;}cos\alpha }>D_{r}+\Delta d,$$

(3) $$n>\frac{1}{\Delta \alpha }{cos}^{-1}\;\frac{l_{i}^{2}+l_{i+n}^{2}-\;{\left(D_{r}+\Delta d\right)}^{2}}{2l_{i}^{\;}l_{i+n}^{\;}},$$

where $l_{i}$, $l_{i+n}$ are the distance measurement signals and represent the right and left ends of the continuous measurement signals, respectively. α is the angle of the two signals ($l_{i}$, $l_{i+n}$) of the robot. $D_{r}$ is the maximum diameter of the robot. The relationship between α and *n* can be expressed by α=n∗Δα. Δα is the resolution of the signal. Δd is a safe distance. Notably, α is greater than 0 and less than π. If α is greater than π, then Equation (2) is surely satisfied.

Frontier type II in Figure 2b is recognized on the basis of the adjacent signals with large differences, as shown in the following formula:

(4) $$\left|l_{j+1}-\;l_{j}\right|*\cos\theta >\;D_{r},$$

where θ is the angle between the direction of $l_{j}$ and the orientation of the robot. In addition, the generated candidate target point based on frontier type II must be reached safely by the robot, as follows:

(5) $$d=2l_{j}sin\frac{\beta }{2}=D_{r}+\Delta d$$

(6) $$l_{j+1,j+2,\ldots ,j+m}>h+\Delta d$$

where β= m·Δα, $l_{j+1,j+2,\ldots ,j+m}$ represents a continuous distance signal from $l_{j+1}$ to $l_{j+m}$, and h is the Euclidean distance between the candidate target point and the robot current location.

The candidate target points are generated at the center of the front edge of the frontier, and the distance between the frontier and candidate target point is *a*, where *a* is a safe distance slightly larger than the maximum radius of the robot.

## 5. Topology–Grid Hybrid Map (TGHM)

**Figure 3:**
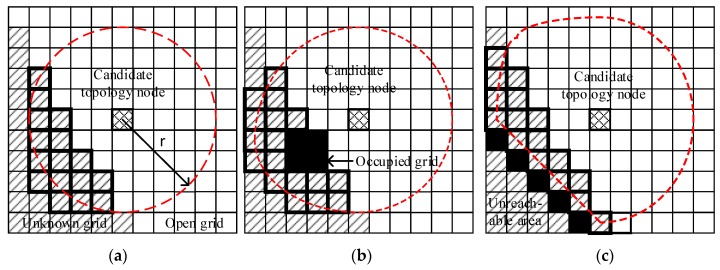
Calculating the information gain around the candidate topology node: (**a**) no obstacle; (**b**) cluster obstacle; (**c**) boundary obstacle.

In the grid map as shown in Figure 3, each grid can fall into one of three classes referring to the related work definition:

1. Open grid: No obstacles.
2. Occupied grid: Obstacles in this grid.
3. Unknown grid: No information in this grid for the robot.

TGHM is the core of the proposed algorithm and is used as follows:

1. The newly generated candidate target points are added to the topology map as candidate topology nodes. When the topology map is updated, each candidate performs a grid traversal within a certain range to calculate the information gain around it. This information gain is used to evaluate the exploration value of the candidate topology node.
2. The topology map records each visited and unvisited nodes. The topological distance between any two topology nodes can be quickly calculated on the basis of the topology map.

The topology node can be represented as

(7) T={Parents,Children},

where Parents and Children are the parent and children nodes of the topology node, respectively. The candidate topology node only has a parent node, which is defined as

(8) $$T_{c}=\left\{\mathrm{Parents},\mathrm{Pose},V\right\},$$

where Pose is the robot’s pose at this node if $T_{c}$ is selected as the next target point. V is the value of the utility function explored at the position. The utility function is defined as follows:

(9) $$V=\;F\left(T\right)=\;N_{unknown}\cdot \exp\left(-\lambda L\left(T\right)\right),$$

where λ is a positive constant and L(T) is the topology distance between the target node T and the current node. The information gain can be calculated on the basis of the number of unknown grids, represented by $N_{unknown}$. The candidate topology node with maximal V is selected as the next target point.

Constant λ is used to weigh the motion cost against the expected information gain. A small λ means that the motion is “cheap” and prioritizes the information gain. When λ → ∞, the motion becomes too expensive that only locations near T are selected. Hence, a small λ leads the robot to perform a quick exploration of the environment before filling in the details. By contrast, a large λ leads the robot to fill in the details consistently while advancing in the environment.

### 5.1. Evaluating Candidate Topology Node on the Grid Map

Exploration based on FastSLAM [34] generally uses occupancy grid maps to show the environment. After the occupancy grid map is constructed, the grid can be divided into three categories, namely, occupied, open, and unknown grids, as shown in Figure 3a. The shadow grid represents the unknown grid, the blank grid represents the open grid, and the mesh shadow grid represents the candidate topology node. The circular dotted line indicates the approximate range of traversal. 

The information gain at a candidate topology node can be expressed by the number of surrounding unknown grids. To calculate the number of unknown grids, the grids are traversed on the basis of the breath-first traversal. The number of traversal grids represented by *N* is limited by the size of the traversal range. The size of the grid traversal range should be based on the actual situation. To calculate the value of *N*, the traversal area is defined as a circular area with the radius of sensor measurement distance. The value of *N* can be limited as follows:

(10) $$N=\frac{\pi r^{2}}{\Delta d^{2}},$$

where Δd is the grid map resolution. The occupied grids are not traversed.

When obstacles exit in the traversal range, its shape can be distorted, as shown in Figure 3b,c. The black grid represents obstacles that block the traverse; thus, the unreachable area will not be counted in the information gain.

### 5.2. Establishing and Updating the Topology Map

Each candidate topology node that does not meet the requirement will be deleted from the topology map. The requirement is that when traversing around each candidate target point, the number of recorded unknown grids ($N_{unknown}$) must be larger than a fixed value ($N_{0}$). The calculation is shown in the following formula, where k represents the serial number of candidate target points:

(11) $$N_{unknown}^{k}>N_{0}.$$

The fixed value is adjusted in accordance with the quality requirements of the map. When the quality requirement is high, the fixed value is small. High quality means that the number of unknown grids on the grid map is low when the exploration is accomplished. 

**Figure 4:**
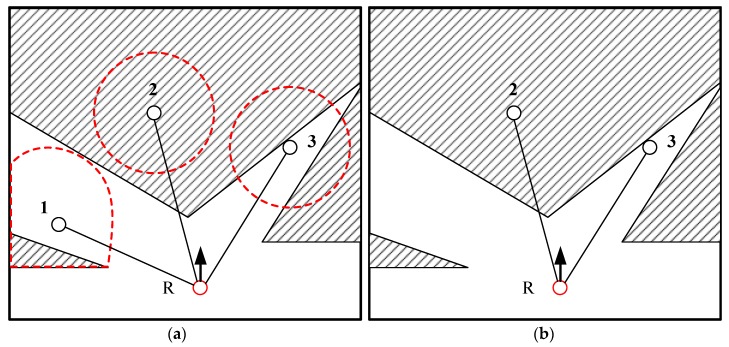
Establishing the candidate topology node: (**a**) generation of candidate topology nodes; (**b**) update of the topology map.

Figure 4 shows the establishment of the candidate topology node. The blank and shaded areas are the freedom and unknown areas, respectively. R is the robot’s current position. In Figure 4a, three candidate target points generated by the geometric rule are represented by 1, 2, and 3, and these points are included in the topology map as candidate topology nodes. The line is the connection line of the topology map. Then, the update of the topology map is started. The circular dotted line represents the traversing range. The information gains, $N_{unknown}^{1,\;2,\;3}$ calculated by Equation (10), were evaluated to filter the redundant candidate topology node by Equation (9). Assuming that point 1 is unqualified and deleted due to the low value of $N_{unknown}^{1}$, only points 2 and 3 with information gains higher than $N_{0}$ are inserted into the topology map as candidate topology nodes, as shown in Figure 4b.

**Figure 5:**
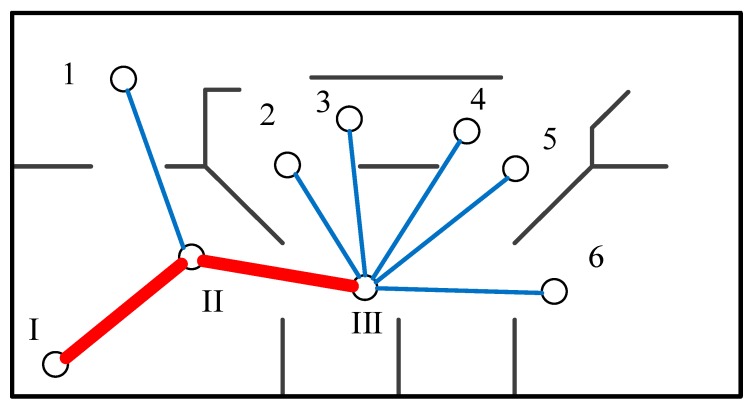
Updating the information gain of the topology map.

When the exploration starts for a while, a small topology map can be generated, as shown in Figure 5. Each target point that has been visited will be used as a topology node (I, II, III). These nodes are connected by thick solid lines. Candidate topology nodes are connected by thin solid lines. 

When one target point is visited, the information gain of each candidate topology node will be updated. If $N_{unknown}<N_{0}$, then a corresponding candidate topology node will be excluded. Under the guidance of this global planning, the mobile robot can quickly determine the next unknown area, thereby greatly reducing repeated exploration and increasing efficiency. When no candidate topology nodes are found, the exploration is over.

## 6. Exploration Based on TGHM Algorithm

**Figure 6:**
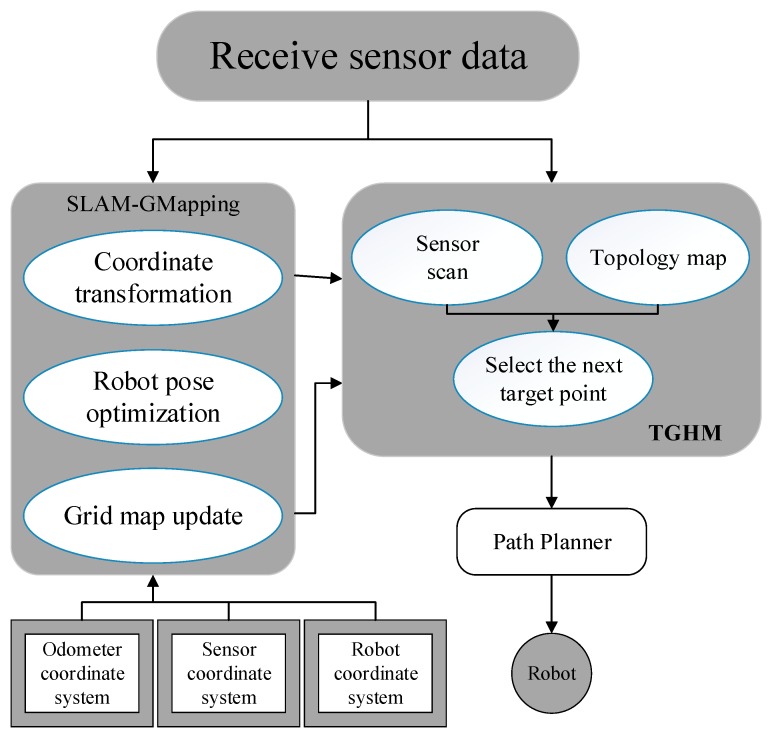
The modular structure of exploration.

Figure 6 presents the modular structure of the proposed exploration algorithm. The function modules are represented by rounded rectangle boxes. SLAM-GMapping [33] is a modular application of the laser SLAM algorithm. The GMapping ROS [35] node contains coordinate transformations, robot pose optimization, and grid map updates. Among them, the coordinate conversion involves the odometer, sensor, and robot coordinate systems. The TGHM node receives data from the sensor, each coordinate, and a grid map. On the basis of the sensor scan, the candidate target points are generated and converted into candidate topology nodes. The candidate topology nodes are evaluated in accordance with the grid and topology maps to select the optimal target point. The speed control command is the output via Move_base [36] that directs the mobile robot to the target point.

Algorithm 1 describes the whole process of the proposed algorithm.
**Algorithm 1:** TGHM Exploration Algorithm**Input:** Laser data *X* generated by LIDAR; Odometer data *O* generated by the mobile robot;**Process:**1:Initialize the topology node set *T_t_* with the origin position *P*_0_: *T*_0_
*=* {*P*_0_};2:Initialize the grid map *M* according to *X* and *O* and the topology map *T* according to *T*_0_;3:Initialize the candidate target point set *C*_0_, the candidate topology node set *N*_0_: *C*_0_, *N*_0_
*=* ∅;4:Initialize *t* as the round number of the exploration: *t* = 1;5:repeat6:  Update *C_t_* with candidate target points generated by geometry rules;7:  if *C_t_*
*≠* ∅8:    Update *N_t_* with filtered candidate target points meet from *C_t_*:*N_t_ = N_t−*1*_*
*∪ C_t_;*9:    Filter the candidate topology nodes according to Equations (10) and (11):      *N_t =_ N_t_\{N_t_}_unqualified_*;10:  else11:    *N_t_ = N_t_*_−1_*;*12:  end if13:  if *N_t_* ≠ ∅14:    Choose the node with the highest value from *N_t_* by Equation (9) as the next target point *P_t_;*15:  else16:    The exploration finishes: return *M* and *T*;17:  end if18:  Motivate the robot towards *P_t_* and update *M* according to *X_t_* and *O_t_;*19:  Turn *P_t_* into a topology node and update *T_t_* accordingly: *T_t_ = T_t_*_−1_ ∪ {*P_t_*}, *N_t_* = *N_t_*\{*P_t_*};20:  Update the topology map *T* with *T_t_;*21:  *t* = *t* + 1;22:until return called**Output**: The established map *M* and the topology map *T*;

The following parts of this section discuss the two main parts of the proposed TGHM algorithm with example experiments to indicate the detailed processes. 

### 6.1. Extracting Candidate Target Points

**Figure 7:**
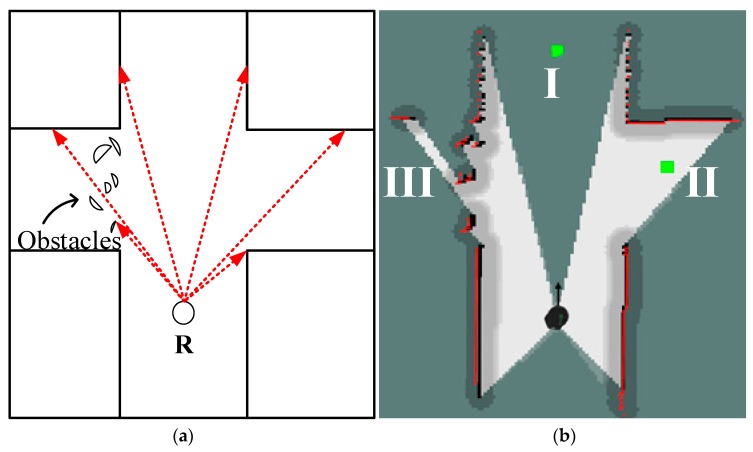
Generating candidate target point experiment: (**a**) sketch map; (**b**) actual effect.

To verify the validity of generating candidate target points on the basis of geometry rules, an example experiment is shown in Figure 7. *R* is the pose of the robot. The red lines in Figure 7a and red frontiers in Figure 7b are the laser ray and laser scanned boundary, respectively. Several obstacles are set on the left side. One laser scan generates two candidate target points as represented by the green point (I, II) and shown in Figure 7b. Points I and II are the points of frontier types I and II, respectively. No candidate target point is in area III because this area is not passable and the laser range distance in this direction does not satisfy Equations (5) and (6). As a result, points I and II are fed to the TGHM algorithm as candidate topology nodes after filtering according to Equations (10) and (11).

### 6.2. Building and Updating the Topology Map

**Figure 8:**
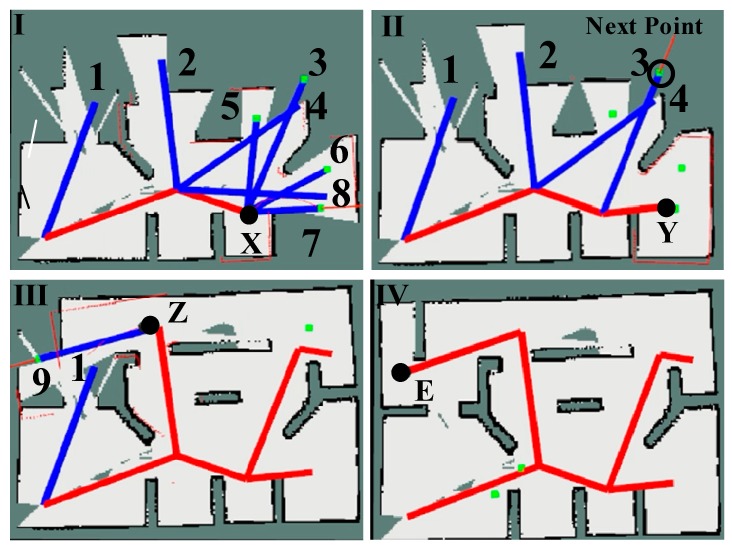
Process of building the TGHM.

To verify the validity of the TGHM algorithm, this study designed a representative experiment to demonstrate the exploration process on the basis of the TGHM algorithm. As shown in Figure 8, the environment size is 10 ×8 m. The visited topology nodes are connected by edges in red and comprise the explored parts of the topology map. The edges in blue connect the valuable candidate topology nodes marked with numbers generated from previous rounds, and the candidate topology nodes in green with edges are generated within the active area, around the current topology node where the robot is. The black object in the figure represents the robot and its position, and the red arrow represents the location and the orientation of the selected next target point in Figure 8II,III.

In Figure 8I, the robot is at the position X and executed two rounds of exploration. The nodes (1, 2, 4, and 8) are generated by the previous rounds with value to be reached afterward if no new candidate target points are generated by the geometry rules. Four candidate topology nodes (3, 5, 6, and 7) are generated currently and candidate topology node (7) with the highest value calculated from Equation (9) is selected as the next target point. In Figure 8II, once the robot reaches the position Y corresponding to the node (7) in (I), all the candidate topology nodes are updated. No new candidate nodes are generated in (II) and the candidate topology nodes (5, 6, and 8) are deleted as their information gains are relatively low. The nodes (1, 2, 3, and 4) are kept temporarily. Then, the TGHM algorithm selects the node (3) with the highest utility value as the next target point. Subsequently, the node (4) is deleted from the topology map and the node (2) is selected as the next point. As shown in Figure 8III, the robot reaches the position Z corresponding to the node (2) and generated one new candidate target node (9). The node (9) is selected as the next target point. Once the robot reaches the node (9), the node (1) is deleted for its low updated value. Figure 8IV presents the TGHM after accomplishing the exploration and the robot reaches the position E corresponding to the node (9). When no candidate topology nodes are generated and all updated topology nodes are visited, the TGHM algorithm suspends the robot’s action and announces the end of exploration.

## 7. Simulations and Experiments

The following simulations and experiments were performed to verify the advantages of the proposed algorithm (TGHM) in the exploration. The simulated laser data operated in the range of (−135°, 135°). The laser’s limit is 5.0 m and the scanning interval is 0.375°. The samples of the laser ray amount to 720. The real-world experiments were performed on a mobile robot with a LIDAR sensor.

### 7.1. Simulations

**Figure 9:**
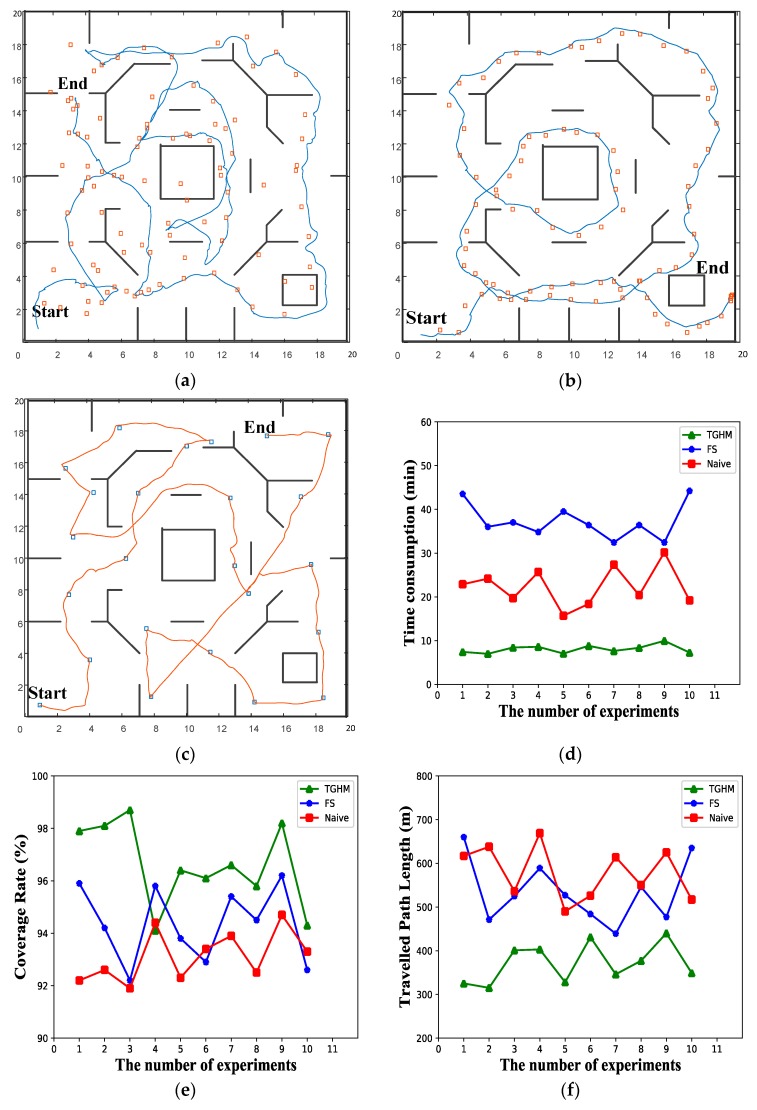
Three types of algorithm for comparison experiment: (**a**) naïve algorithm; (**b**) forward simulation (FS) algorithm; (**c**) TGHM algorithm (ours); (**d**) line chart of exploration time consumption; (**e**) line chart of the coverage rate of the established map; (**f**) line chart of the traveled path length.

A series of simulations were conducted to confirm the advantages of the TGHM algorithm in exploration efficiency. The simulation map is a 20 ×20 m map, as shown in Figure 9. Three algorithms were used in the simulations, namely, TGHM, FS [26], and Naïve [19]. Each experiment for the three algorithms was simulated 10 times to ensure reproducibility.

The simulated robot can turn on the spot and is controlled by applying linear and angular velocity. The maximum linear velocity is 1 m/s, and the maximum angular velocity is 1 rad/s. The robot was equipped with a laser range finder with a maximum range of 5.0 m and a scan range from −135° to 135°.

Figure 9 shows the trajectories obtained by the three algorithms. The square blocks are target points generated during the movement. For the naïve algorithm, the target point is generated at 1 Hz. The robot moves to the target point regardless if the last point has been reached. Therefore, many target points are unvisited, as shown in Figure 9a. In Figure 9b, the FS algorithm takes relatively more time for each calculation, which limits the distance of the simulation planner. In comparison with the first two algorithms, the TGHM algorithm calculates the least target points, as shown in Figure 9c, and its time spent in the lowest, as shown in Figure 9d. For the quality of the established map, all three algorithms managed to achieve a high coverage rate in the simulation, as shown in Figure 9e. The traveled path length data is also plotted in Figure 9f, which indicates the proposed TGHM algorithm is able to finish the exploration within a relatively short path.

Table 1 shows the benchmark results from the simulation experiments, where columns 1 to 10 indicate the results from 10 simulations for each algorithm. Since all three algorithms are capable of established the full environment map, the time consumption, the number of target points, the map coverage rate, and the traveled path length are the main indicators. The time consumption directly reflects the efficiency of the exploration algorithm and the number of target points related to the amount of the calculation and the mean speed for the robot movement along with the traveled path length. Once the environment map is established, the coverage of the map against the ground truth influents the robot navigation performance.

The results from Table 1 show that the time cost of the TGHM algorithm and the average number of target points are the lowest among the tested algorithms. The coverage rate among the benchmarked algorithms is close. The main reason comes from the following aspects:

1. The generation of the candidate target points is based on the environment geometry rules. The valuable candidate target points can be quickly obtained, avoiding iterative calculation. Thus, the time consumption for each round of exploration is dramatically reduced.
2. The incremental caching TGHM is used to provide global planning for the entire exploration task. The topology map is updated for each round and only keeps the valuable nodes necessary for the coverage of the environment map.
3. The TGHM algorithm has the lowest number of target points compared with the other two algorithms. As a result, the time consumption carried by the number of all target points is decreased, which greatly improves exploration efficiency.
4. The traveled path length is relatively low for the proposed TGHM algorithm because the less amount of target points lead to better-planned robot motions with higher average speed and less redundant movement under the slight difference in map coverage rate.

**Figure 10:**
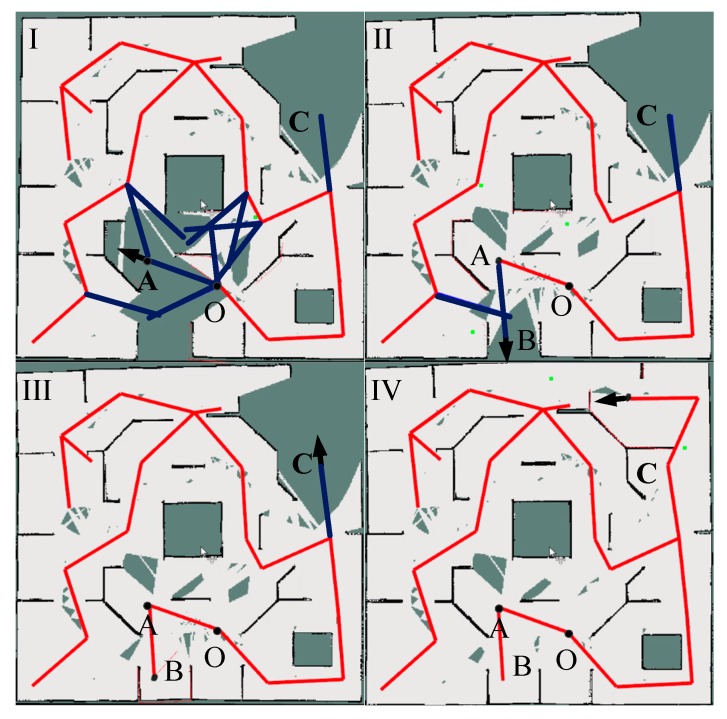
Exploration process: (**I**) Map established after rounds of exploration; (**II**) Map after the robot reached point A; (**III**) Map after the robot reached point B; (**IV**) Final map after the exploration; Point A is a target point in (**I**) and a visited topology node in (**II**–**IV**); Point B is a target point in (**II**) and a visited topology node in (**III** and **IV**); Point C is a candidate topology node in (**I**–**III**) and a visited topology node in (**IV**); Point O is a visited topology node in (**I**–**IV**).

Figure 10 shows the autonomous exploration process of a robot based on the TGHM algorithm. Thin red lines connect the visited topology nodes. Each candidate topology node is connected to their parent topology nodes by edges in red for visited ones and in blue for unvisited ones. As previously mentioned in Equation (10), the requirement of keeping topology nodes is that the information gain of each candidate topology node must be larger than a fixed value. The black arrows indicate the position and orientation of the current target node. Figure 10I,II show the robot and the map after rounds of exploration when the mobile robot tends to move from node O to point A. After the grid and topology maps are updated accordingly, topology node A is added, and other candidate topology nodes with substandard information gain are deleted. Once the robot reaches the position of node A, the next round begins and the new topology node B is selected as the next target point as before. In Figure 10III, the mobile robot moves from point A to point B and does not generate any new candidate topology node that has sufficient information gain, indicating that this active area is well explored. Considering the topology path cost and information gain, candidate topology point C is selected as the next target point. Figure 10IV shows the final effect of automatic exploration. The topology map can be helpful for subsequent navigation.

### 7.2. Experiments

**Figure 11:**
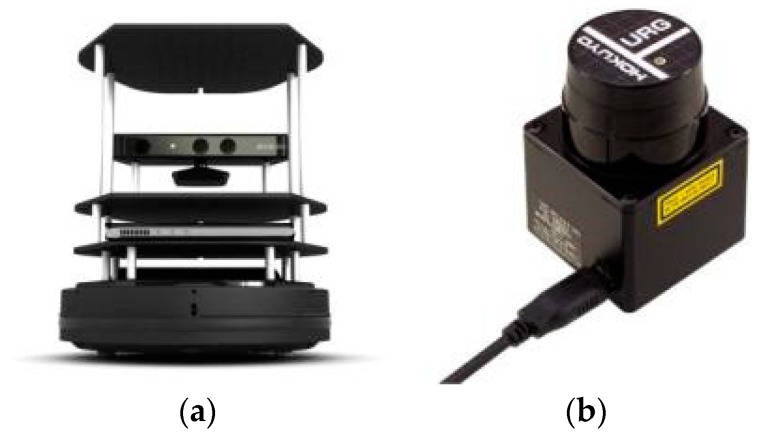
Experiment apparatus: (**a**) Turtlebot; (**b**) URG-04LX-UG01 laser scanner.

Experiments were further performed on a Turtlebot II robot platform [37] equipped with a URG-04LX-UG01 [38] laser scanner (LIDAR), as shown in Figure 11. The laser scanner has a scanning range of 240° with 0.352° angular resolution and a detectable range from 20 to 5600 mm with a 1 mm resolution. Its measuring accuracy is +/−30 mm from 0.06 to 1 m and 3% of the detected distance from 1 to 4 m. The scanner is connected to a computing unit and mounted horizontally on the top of the Turtlebot.

**Figure 12:**
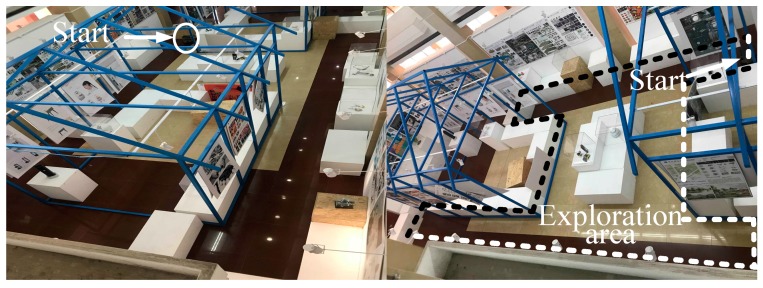
Experimental environment.

Figure 12 shows the school building of East China University of Science and Technology. The size of the environment is 6×12 m. The dotted area is the scope of exploration of the mobile robot. The task of the mobile robot is to build a map for navigation without artificial manipulation. The position indicated by the red arrow is the start position of the robot. Similarly, the exploration task was performed by using three algorithms (TGHM, FS, and Naïve). 

**Figure 13:**
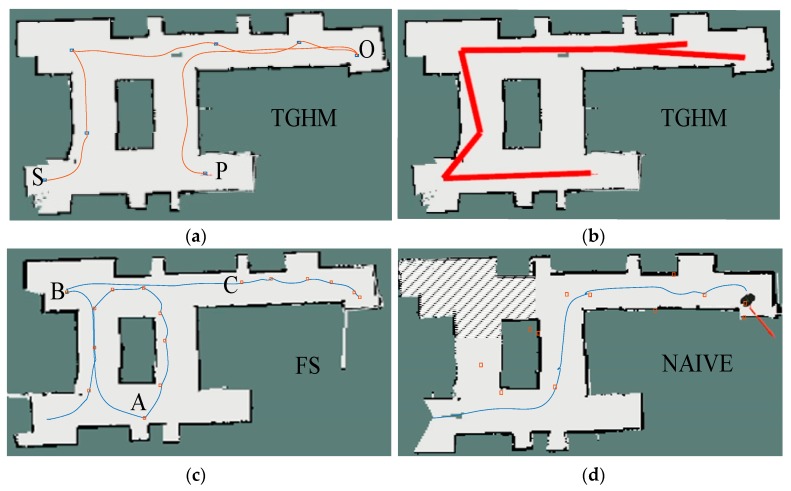
Trajectory graph: (**a**) TGHM; (**b**) grid–topology hybrid map; (**c**) FS; (**d**) Naïve.

Figure 13 shows the robot trajectory using three algorithms. The square points represent the target points generated by algorithms during the exploration. In Figure 13a, point P was generated as a candidate topology node in the first round when the robot started exploration at point S. When the mobile robot was at point O and the algorithm did not generate candidate topology nodes that met the requirements, point P became the next target point from the appending candidate topology node. When the robot reached point P, the exploration is finished and the topology map was updated, as shown in Figure 13b. Figure 13c shows the trajectory of robot exploration based on the FS algorithm. The result was similar to the simulation results, except when the FS algorithm did not generate the target point in the FS process, the Naïve algorithm was called as a supplement. From point A to point B, from point B to point C, the Naïve algorithm was called in the FS algorithm due to having no target point generated by the FS algorithm.

Figure 13d shows the trajectory of exploration based on the Naïve algorithm. The Naïve algorithm failed to explore the whole environment map, thus the robot traveled a relatively short path. The shadow in the figure indicates the part where the Naïve algorithm did not explore. But given the disorder of the generated target points and unreachable points, the time consumption was as high as FS. 

Table 2 demonstrates the results of the real-world experiments, where columns 1 to 3 correspond to the three experiments for each algorithm. As shown in Table 2, the naïve algorithm only established part of the environment map with a coverage rate of 74.87%. The proposed TGHM algorithm and the benchmarked FS algorithm managed to achieve a high coverage rate with a small difference. However, in terms of time consumption and the traveled path length, the TGHM algorithm demonstrated higher efficiency. The geometry-rules candidate generation method and the topology–grid hybrid map produced the low amount of target points, which lead to more efficient robot movements and a more timesaving exploration.

### 7.3. Discussion

In this section, we discuss the results of the simulations and experiments. Both simulations and experiments indicated that the proposed algorithm with an incremental caching topology–grid hybrid map (TGHM) achieved a more efficient exploration when maintaining the high coverage rate of the established map. The geometry-rules method, as described in Section 4, accelerates the candidate point generation first. Then the topology–grid hybrid map filters the redundant candidate point and arranges the nodes in a manner of maximum information gain. The topology map updates and removes the invaluable nodes in each round of exploration to keep the robot motion efficient.

Notice that the difference of traveled path length between the benchmarked algorithms is smaller than the difference of exploration time. This is because the fewer target points generated according to geometry rules lead to the faster movement of the robot with a limited upper speed.

## 8. Conclusions

This study proposes an algorithm based on an incremental caching TGHM to solve the robot’s automatic exploration problem. In comparison to the FS and Naïve algorithms with low mapping efficiency, the proposed TGHM algorithm selects the target point by considering information gain and topology path cost. Thus, the total exploration time is greatly reduced. The mobile robots can then explore the environment with enhanced stability and increased efficiency. The established map can be efficiently traversed and the entire grid map can be generated.

The advantages of the proposed algorithm in exploration efficiency have been demonstrated through simulations and experiments. Future work falls into the following two aspects. First, the generation of candidate target points will be extended from two to three dimensions, which can help mobile robots avoid many realistic restrictions. Second, the topology map will be further optimized. For example, two near topology nodes can be combined into one. A further complicated structure will be used to represent the topology map, wherein a loop can be formed.

## Figures and Tables

**Table 1:** Units for magnetic properties.

Algorithm		1	2	3	4	5	6	7	8	9	10	Avg
TGHM	Time (min)	7.41	6.98	8.42	8.58	7.04	8.81	7.63	8.36	9.96	7.26	8.045
	Target points	24	22	28	29	22	30	25	28	32	22	26
	Coverage (%)	97.9	98.1	98.7	94.1	96.4	96.1	96.6	95.8	98.2	94.3	96.62
	Travelled Path (m)	325	315	401	403	328	431	346	377	440	349	371.5
FS	Time (min)	43.5	36.0	37.0	34.8	39.5	36.4	32.4	36.4	32.4	44.2	38.25
	Target points	159	132	135	127	145	133	119	162	152	136	140
	Coverage (%)	95.9	94.2	92.2	95.8	93.8	92.9	95.4	94.5	96.2	92.6	94.35
	Travel Path (m)	660	471	525	589	527	484	439	546	477	635	535.3
Naïve	Time (min)	22.9	24.2	19.7	25.7	15.7	18.4	27.4	20.4	30.2	19.2	22.41
	Target points	162	178	153	188	122	149	193	150	215	150	165
	Coverage (%)	92.2	92.6	91.9	94.4	92.3	93.4	93.9	92.5	94.7	93.3	93.12
	Travelled Path (m)	617	638	536	669	490	526	614	550	625	517	578.2

**Table 2:** Exploration time of each algorithm.

Algorithm		1	2	3	Avg
TGHM	Time (min)	2.51	2.46	2.78	2.58
	Target points	7	7	8	7
	Coverage (%)	98.9	96.4	99.1	98.13
	Travel Path (m)	72.39	67.78	77.52	72.53
FS	Time (min)	4.52	4.79	5.06	4.79
	Target points	16	17	18	17
	Coverage (%)	97.5	95.5	96.7	96.56
	Travel Path (m)	103.05	88.44	112.25	101.25
Naïve	Time (min)	4.47	5.84	5.23	5.18
	Target points	12	15	13	13
	Coverage (%)	69.9	79.1	75.6	74.87
	Travel Path (m)	53.79	72.62	66.52	64.31
